# Interplay of normative beliefs and behavior in developmental patterns of physical and relational aggression in adolescence: a four-wave longitudinal study

**DOI:** 10.3389/fpsyg.2014.01146

**Published:** 2014-10-15

**Authors:** Barbara Krahé, Robert Busching

**Affiliations:** Department of Psychology, University of PotsdamPotsdam, Germany

**Keywords:** physical aggression, relational aggression, aggressive norms, adolescence, gender, longitudinal study, Germany

## Abstract

In a longitudinal study with *N* = 1,854 adolescents from Germany, we investigated patterns of change and gender differences in physical and relational aggression in relation to normative beliefs about these two forms of aggression. Participants, whose mean age was 13 years at T1, completed self-report measures of physically and relationally aggressive behavior and indicated their normative approval of both forms of aggression at four data waves separated by 12-month intervals. Boys scored higher than did girls on both forms of aggression, but the gender difference was more pronounced for physical aggression. Physical aggression decreased and relational aggression increased over the four data waves in both gender groups. The normative acceptance of both forms of aggression decreased over time, with a greater decrease for the approval of physical aggression. In both gender groups, normative approval of relational aggression prospectively predicted relational aggression across all data waves, and the normative approval of physical aggression predicted physically aggressive behavior at the second and third data waves. A reciprocal reinforcement of aggressive norms and behavior was found for both forms of aggression. The findings are discussed as supporting a social information processing perspective on developmental patterns of change in physical and relational aggression in adolescence.

## INTRODUCTION

A growing body of longitudinal research has examined the developmental trajectories of aggressive behavior through childhood and adolescence (see Farrington, 2007; Krahé, 2013, for reviews). This research has yielded evidence of an age-normative decline of aggression as children get older, despite the fact that some children show persistently high or increasing levels of aggressive behavior (Loeber and Stouthamer-Loeber, 1998; Moffitt, 2007). In understanding these developmental pathways, it has turned out to be fruitful to expand the traditional focus on physical aggression to include relational aggression as another modality in which aggressive behavior may be expressed. In particular, this focus has been influential in the study of gender differences in aggression, as relational aggression has been conceptualized as more consistent with female gender norms than physical aggression (Richardson and Hammock, 2007).

Physical aggression refers to behaviors intended to cause physical harm to the target person, whereas relational aggression consists of behaviors intended to cause harm by manipulating and damaging the target person’s peer relationships (Crick and Grotpeter, 1995). Thus, the two forms of aggression share the same underlying motivation but differ with regard to the “vehicles of harm” (Crick et al., 2007). Although different in form, physical and relational aggression may be equally hurtful (Crick et al., 1996). Instead of using the concept of relational aggression, other authors have preferred the terms “indirect aggression” (e.g., Cleverley et al., 2012) or “social aggression” (e.g., Underwood, 2003). Archer and Coyne (2005) concluded from their review of the literature that “there are very few differences between indirect, relational, and social aggression in terms of the actions involved, their development, sex differences, and consequences” (p. 225), and the constructs converge on a common theme, which is the harming of interpersonal relationships (Card et al., 2008; Warren et al., 2011). This aspect is best captured in our view by the term “relational aggression,” which is therefore adopted for the present paper, but the other terms will be referred to as they are used by the authors of the respective studies. Past research has focused primarily on studying differences between physical and relational aggression in middle childhood (see Crick et al., 2007, for a summary), and far fewer studies are available to date that have extended the analysis of the two forms of aggression to adolescence (e.g., Werner and Nixon, 2005; Underwood et al., 2009; Cleverley et al., 2012).

Despite considerable variability at the individual level, the age-normative pattern in the development of physical aggression has been found to be a decline from middle childhood onward (Loeber and Hay, 1997). The developmental pattern of relational aggression has been studied less widely and seems to be less clear (Underwood et al., 2001). There is some evidence that through childhood and early adolescence relational aggression increases with age (see Vaillancourt, 2005, for a review), but little is known about changes in relational aggression beyond early adolescence. A cross-sectional study with adolescents in 6th, 7th, and 9th grade found that girls’ indirect aggression, measured through peer nominations, was higher in the older cohorts, but no corresponding increase was found for boys (Owens and MacMullin, 1995). Based on the theoretical proposition that relational aggression requires more social skills than physical aggression (Kaukiainen et al., 1999) and given the increasing importance of peer relationships in adolescence, one might expect an increase in relational aggression in the course of adolescence. The present study investigated this proposition.

Interest in relational aggression has been prompted by the recognition that the available evidence that males are more aggressive than females is largely based on studies examining physical aggression, which is more in line with male than with female gender role socialization (Smith et al., 2010). Expanding the scope of aggression to include forms that are more compatible with the female gender role has facilitated a more comprehensive appraisal of the issue of gender differences in aggressive behavior. There is conclusive evidence that boys are more physically aggressive than are girls, based on different operationalizations of physical aggression, such as self-reports, peer nominations, and teacher reports (Archer, 2004). At the same time, evidence is mixed with regard to gender differences in relational aggression and varies as a function of methodology (parent, peer-, and self-reports as well as behavioral observation; Archer and Coyne, 2005). Meta-analytic studies on relational aggression confirmed that gender effects were heterogeneous across informants and concluded that gender differences were negligible overall (Card et al., 2008; Scheithauer et al., 2008).

A study including children aged 7–10 from nine countries found consistently higher reports of physical aggression among boys, but no gender difference in relational aggression (Lansford et al., 2012). Longitudinal research confirmed this pattern of results by showing that boys scored consistently higher than did girls on measures of physical aggression, but boys and girls did not differ in their level of relational aggression (Cleverley et al., 2012; Kawabata et al., 2012; Tseng et al., 2013). These studies provide little evidence of gender differences in the propensity to show relationally aggressive behavior in childhood and early adolescence. One of the few studies of older adolescents by Prinstein et al. (2001) also found significant gender differences in physical, but not relational aggression in their sample of 9th to 12th graders, but they did not examine any age effects within their sample.

Even less evidence is available about changes of gender differences in relational aggression in the course of childhood and adolescence beyond a comparison of studies including different age cohorts. Smith et al. (2010) used a cross-sectional multi-cohort design covering grades 3, 5, 7, and 9 and assessed physical and relational aggression through peer reports. They found that gender differences in both physical (boys scoring higher than girls) and relational (girls scoring higher than boys) aggression were greater in the older grade cohorts, but only when controlling for the respective other form of aggression. However, their study could not identify changes in the pattern of gender differences over time. In the present study, the interaction of time, aggression form, and gender was studied in a longitudinal design to examine the proposition that with boys’ increase in relational aggression in the course of adolescence, gender differences in relational aggression would diminish.

One reason for the normative decline of physical aggression as children grow older is the learning of social norms that regulate the performance of aggressive behavior. Socio-cognitive explanations of aggression assign a key role to normative beliefs about aggression that guide individuals’ information processing and behavioral choices regarding aggressive behavior (Huesmann and Guerra, 1997; Huesmann, 1998; Fontaine and Dodge, 2009). If “normative beliefs serve to regulate corresponding actions by prescribing the range of allowable and prohibited behaviors” (Huesmann and Guerra, 1997, p. 409), it follows that such beliefs should predict behavior to the extent that there is a match between the contents of the normative beliefs and the range of behaviors in question. Normative beliefs about the appropriateness of physical aggression should be more closely related to physical aggression than to other forms of aggression, such as relational aggression, and normative beliefs about relational aggression should be more closely related to measures of relationally as opposed to physically aggressive behavior. In line with this proposition, Werner and Nixon (2005) showed in a study of 7th and 8th grade adolescents that the link between normative beliefs and behavior was specific to the form of aggression considered, such that normative approval of relational aggression predicted relationally but not physically aggressive behavior over a 10-week period, whereas normative approval of physical aggression predicted physically, but not relationally, aggressive behavior.

Social information processing models of aggressive behavior have assumed a reciprocal influence of social cognitive appraisals and decisions relating to aggression and actual behavior. In their “individual systems model of response evaluation and decision-making” (RED), Fontaine et al. (2008) proposed a bidirectional influence between aggressive behavior and decision-making processes about aggressive responses. Normative beliefs can be seen as playing an important role in these social decision-making processes (Huesmann, 1998). While there is ample support for the path from normative beliefs to aggression, evidence is less widely available and also less consistent regarding the reverse path from aggressive behavior to normative beliefs. Huesmann and Guerra (1997) found that aggressive behavior predicted subsequent normative approval of aggression in their younger cohort of 2nd graders, but not in the older cohort of 5th graders. Werner and Hill (2010) did not find evidence of a path from aggressive behavior to normative beliefs. However, neither of these studies included more than two data waves, so they were unable to follow the reciprocal relationships of normative beliefs and aggressive behavior over time. Fontaine et al. (2008) examined the reciprocal paths from aggressive response evaluation and antisocial behavior over five points in time from grade 7 to grade 12 (ages 13–17). In support of their “individual systems” model, they found significant paths from grade 7 antisocial behavior to grade 8 response evaluation and from grade 8 response evaluation to grade 9 antisocial behavior, with a continuation of this pattern up to grade 12. Although their study did not focus specifically on normative beliefs, participants’ social outcome expectancy in terms of how an aggressive response to a hypothetical scenario would be evaluated by others was included as one aspect of the response evaluation and decision measure. In the present study, the reciprocal influences of normative beliefs as social cognitions involved in response decision-making and aggressive behavior were studied in a similar age group of adolescents in Germany. In particular, we sought to demonstrate that physical and relational aggression influence, and are influenced by, corresponding normative beliefs that are specific to the respective forms of aggression.

Based on the theoretical considerations and previous findings reported above, the current study was designed to examine gender differences and patterns of development of physical and relational aggression in adolescence in relation to normative beliefs. Agewise, the present analysis started where most previous studies on the link between normative beliefs and physical as well as relational aggression have ended by following participants from early to middle adolescence (age 13 to age 16) over four data waves. Based on the theorizing that relational aggression increases in line with increasing social-cognitive skills (Kaukiainen et al., 1999), we examined the proposition that relational aggression would increase in the course of the 3-year period, whereas physical aggression was expected to decline over time, because the increasing social skills promote awareness of the normative sanctioning of physical aggression. Also in line with previous evidence, mostly from North America, we expected to find larger gender differences in physical aggression than in relational aggression. In addition, we sought to further elucidate the role of normative beliefs by showing that each of the two forms of aggression is linked to specific normative beliefs and that norms and behavior mutually reinforce each other over time.

Our predictions were specified in five hypotheses:

*Hypothesis 1*: In the age-normative pattern of development, aggressive behavior changes its expression in the course of adolescence. Physical aggression decreases in favor of relational or indirect forms of aggression that are less visible and less likely to be sanctioned.

*Hypothesis 2*: Boys are consistently more physically aggressive than girls throughout adolescence, whereas the gender difference is small or non-existent with regard to relational aggression.

*Hypothesis 3*: Boys show a greater acceptance of aggression than do girls, with the difference being greater for the approval of physical as compared to relational aggression.

*Hypothesis 4*: Normative beliefs are specific to the form of aggression, with normative approval of relational aggression being more closely related to relational than to physical aggression, and normative approval of physical aggression being more closely related to physical than to relational aggression.

*Hypothesis 5*: Normative beliefs about aggression not only influence aggressive behavior, but aggressive behavior also shapes the normative approval of aggression, resulting in a mutually reinforcing cycle that contributes to the continuity of aggressive behavior over time.

## MATERIALS AND METHODS

### PARTICIPANTS

A total of *N* = 1,854 secondary school students (892 male, 962 female) from 81 classes in 14 secondary schools in Berlin took part in a longitudinal study that covered four data waves separated by 12-month intervals. Participants were in 7th and 8th grade at T1, with a mean age of 13.3 years (SD = 0.87, range: 11–16 years). The mean age at T4 was 16.3 years (SD = 0.92). Of the total sample, 71.0% of the participants were German nationals, 9.5% were Turkish nationals, 10.1% had dual nationality of German and another country, the remaining 9.3% came from a range of different countries. However, nationality alone is not a good indicator of ethnic background, as many youth with a migration background hold German passports. Therefore, a multi-indicator variable of ethnic background was created based on nationality, mother tongue, and language spoken at home. Participants were assigned to the non-German ethnic background group if they met at least one of the three criteria: non-German nationality, non-German mother tongue, or language other than German spoken at home. By this definition, 42.9% of participants were assigned to the non-German ethnic background group, and ethnic background was considered as a covariate in the analyses reported below. All school types in Berlin’s three-tier secondary school system (Hauptschule, Gesamt-/Realschule, Gymnasium), varying in academic orientation, were represented in the sample.

Participants were included in the sample if they had attended at least two of the four data waves. Within this sample, participation rates were *N* = 1,312 at T1 (642 male), *N* = 1,581 at T2 (762 male), *N* = 1,489 at T3 (714 male), and *N* = 1,062 at T4 (487 male). A further 823 participants who were only present for one testing session were not included in the analysis. These participants were evenly distributed across the four data waves (215 were present only at T1, 229 only at T2, 169 only at T3, and 209 only at T4). The relatively high dropout rate from T3 to T4 is explained by the fact that some participants in the older cohort had finished school by that time. They were in 8th grade at T1 and consequently in 10th grade at T3, and the secondary school system in Berlin is organized such that the less academically oriented types of school finish after 10th grade. Therefore, these participants could no longer be reached in the class-based testing sessions at T4, and due to data protection constraints, we were unable to follow them up after they had left school. In the analyses, this dropout is accounted for by including school type as a covariate in combination with Full Information Maximum Likelihood estimation for handling missing data.

Approval for all parts of the study was obtained from the Ethics Committee of the authors’ university and the school administration in Berlin. Active consent was obtained from all students. In addition, parental consent was obtained for participants under the age of 14, in line with regulations for school-based research in Berlin. All data were collected by trained project staff during normal class hours.

### MEASURES

#### Aggressive behavior

Aggressive behavior was measured by a 10-item self-report instrument based on Möller and Krahé (2009), asking participants to report how often they had shown the respective behavior toward a peer in the past six months on a scale from 0 (never) to 4 (very often). Five items addressed physical aggression (e.g., “I have pushed another person”; “I have hit another person”), and five items addressed relational aggression (e.g., “I have excluded someone from our group”; “I have spread gossip about people I don’t like”). The five physical aggression items were taken from Björkqvist et al. (1992; two items) and Möller and Krahé (2009, three items), and the five relational aggression items came from Möller and Krahé (2009, two items) and Archer and Coyne (2005, three items). The two-dimensional structure was confirmed through factor analysis (Krahé and Möller, 2010). As shown in **Table 1**, internal consistencies of both measures were good at all four data waves, ranging from α = 0.81 to 0.88 for the physical aggression measure and from α = 0.75 to 0.78 for the relational aggression measure.

**Table 1:** Means of physical aggression, relational aggression, and aggression-related norms.

	Physical aggression (five items, range 0–4)				Relational aggression (five items, range 0–4)				Normative approval of physical aggression (two items, range 0–3)				Normative approval of relational aggression (three items, range 0–3)			
	α	Total	Boys	Girls	α	Total	Boys	Girls	α	Total	Boys	Girls	α	Total	Boys	Girls
T1	0.81	0.65	0.88	0.42	0.78	0.63	0.71	0.55	0.89	0.70	0.96	0.45	0.74	0.98	1.08	0.90
T2	0.82	0.66	0.89	0.44	0.75	0.64	0.74	0.55	0.89	0.62	0.87	0.39	0.73	0.92	1.02	0.82
T3	0.88	0.66	0.97	0.37	0.75	0.70	0.78	0.63	0.86	0.47	0.63	0.32	0.79	0.78	0.83	0.72
T4	0.86	0.53	0.77	0.31	0.77	0.68	0.75	0.62	0.81	0.45	0.63	0.28	0.72	0.87	0.92	0.82

*Total *N* = 1,854; boys *n* = 892, girls *n* = 962. All gender differences are significant at *p* < 0.01.*

#### Normative acceptance of aggression

The normative acceptance of aggression was measured with a vignette describing a provocation scenario based on Möller and Krahé (2009). The vignette read as follows:

Imagine you are extremely angry with one of your classmates because he/she treated you in a mean and unfair way in front of others that morning. After school you meet the person again, and this time the two of you are alone. Immediately he/she starts quarreling with you again, saying nasty things.

Participants were presented with the appropriate version referring to a same-sex peer and were asked to indicate how acceptable they would find each of five responses in that situation. Two responses represented physical aggression (e.g., “to kick and push him/her”), and three responses reflected relational aggression (e.g., “to spread rumors about him/her”). Responses were made on a scale ranging from 0 (not at all ok) to 3 (totally ok). The two-dimensional structure was demonstrated by a confirmatory factor analysis (χ^2^ (df = 4) = 22.41, *p* < 0.001, CFI = 0.99, TLI = 0.98, SRMR = 0.03, RMSEA = 0.06, 95% CI [0.04; 0.08]). All items loaded significantly on their respective factor. The two subscales had good internal consistencies at each data wave, ranging from α = 0.81 to 0.89 for the physical norm scale and from α = 0.72 to 0.79 for the relational norm scale (see **Table 1**).

#### Plan of analysis

The predicted patterns of change in aggressive behavior and in the normative approval of the two forms of physical and relational aggression were examined in two latent intercept-slope analyses in which time and form of aggression were included as within-subjects variables and gender was included as a between-subjects variable. In addition, year cohort, school type, and ethnic background were included as covariates. The specificity of physical and relational normative beliefs as predictors of physical and relational aggression, respectively, was tested through partial correlations between one facet of norms and behavior controlling for the respective other facet. The reciprocal influence of normative beliefs and behavior for each of the two aggression forms was examined in a cross-lagged panel analysis in which gender differences were tested through multi-group models. Missing data as well as the non-normality of the variables were handled by using a robust Full Information Maximum Likelihood estimator implemented in Mplus (Muthén and Muthén, 2012). To take care of the non-independence of the participants from the same class, the standard errors were corrected using the sandwich estimator (Muthén and Satorra, 1995). The indirect effects were calculated using parametric bootstrapping, as suggested by Hayes and Scharkow (2013).

## RESULTS

The means of the two aggression measures and the normative belief scores at each of the four data waves, not considering any covariates, are presented in **Table 1** both for the total sample and the two gender groups. Gender differences in the mean scores were calculated estimating latent slopes models which were also used to investigate Hypothesis 1. Boys scored significantly higher than did girls on all measures of aggression and normative beliefs.

**FIGURE 1:**
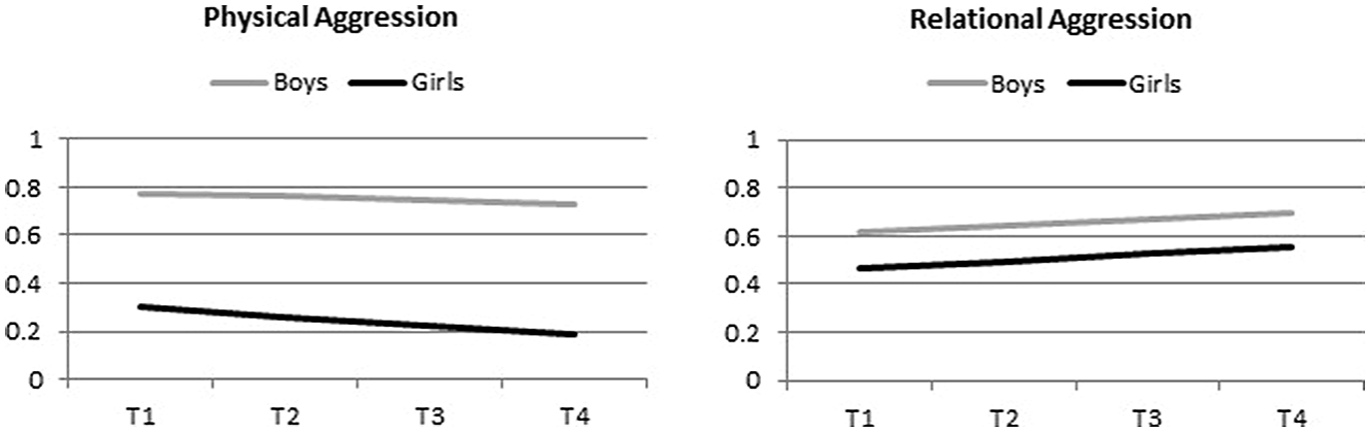
**Changes in physical and relational aggression over time by gender (controlled for year cohort, ethnic background, and school type).** Scale range: 0–4.

In support of Hypothesis 1, a latent intercept-slope analysis yielded a significant interaction of time and form of aggressive behavior, *b* = 0.03, *p* < 0.001. As shown in **Figure 1**, physical aggression decreased and relational aggression increased from T1 to T4 in both gender groups. In addition, a significant main effect of gender was found, indicating that boys scored higher than did girls across time and forms of aggression, *b* = 0.16, *p* < 0.001. This gender main effect was qualified, however, by a significant interaction with aggression form. In line with Hypothesis 2, it was found that the gender difference was larger for physical than for relational aggression, *b* = -0.08, *p* < 0.001. Within-gender comparisons showed that, for boys, the mean for physical aggression was significantly higher than the mean for relational aggression. The reverse pattern was found for girls.

The main effects of time and aggression form, the interaction of time and gender, and the three-way interaction of time, aggression form, and gender were non-significant. Of the covariates, only school type showed a significant main effect, with participants from the more academically oriented school type reporting less aggressive behavior.

**FIGURE 2:**
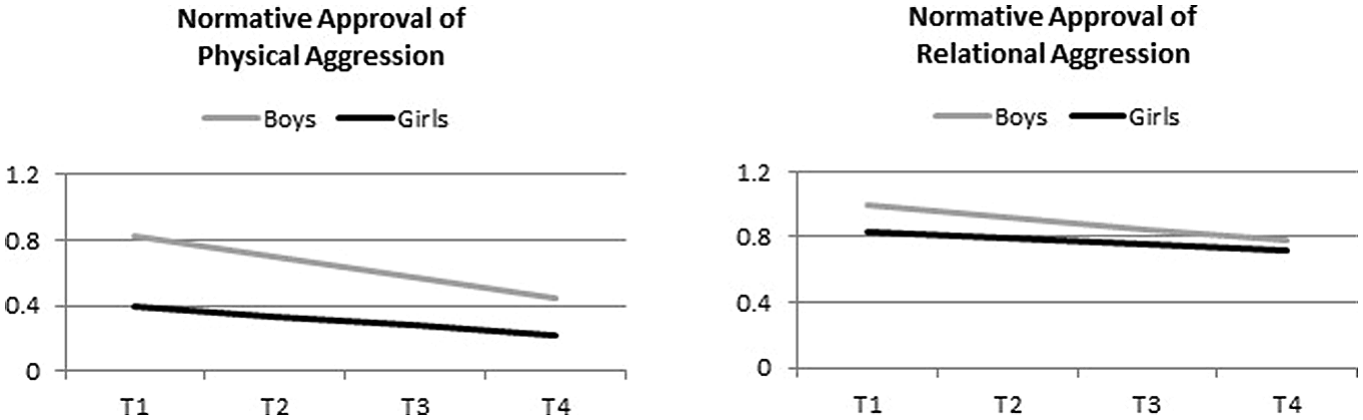
**Changes in the normative approval of physical and relational aggression over time by gender (controlled for year cohort, ethnic background, and school type).** Scale range: 0–3.

A parallel latent intercept-slope analysis was conducted to examine the effects of time, aggression form, and gender on the normative approval of aggression. This analysis yielded a main effect of aggression form, indicating that approval of physical aggression was lower than approval of relational aggression, *b* = 0.15, *p* < 0.001. Moreover, a significant gender effect was found, *b* = 0.15, *p* < 0.001, indicating that boys were more accepting of aggression than were girls. These effects were qualified, however, by a significant interaction of gender and aggression form on normative beliefs, *b* = -0.07, *p* < 0.001, supporting Hypothesis 3. As displayed in **Figure 2**, boys were more approving than were girls of physical but not of relational aggression. The main effect of time was also significant, *b* = -0.07, *p* < 0.01, reflecting a decrease in the normative acceptance of aggression over the four data waves. In addition, a significant time by gender interaction was found, *b* = -0.03, *p* < 0.01, indicating that boys’ normative approval of aggression decreased more than did girls’. Finally, a significant interaction of time and aggression form (physical, relational) indicated that the decrease was larger for the approval of physical as opposed to relational aggression, *b* = 0.02, *p* < 0.01. The three-way interaction of time, gender, and aggression form was non-significant, and none of the covariates (year cohort, school type, and ethnic background) significantly predicted the normative approval of aggression. The estimated trajectories for boys and girls are shown in **Figure 2**.

In Hypothesis 4, we proposed that the two forms of aggression would be more closely linked to the corresponding than the non-corresponding norm facet. The partial correlations computed to test this prediction are presented in **Table 2**. Although most of the partial correlations were significant, the associations were stronger between the corresponding measures of norms and behavior (physical aggression with physical norms; relational aggression with relational norms) than the non-corresponding associations (physical aggression with relational norms; relational aggression with physical norms). Since Mplus does not facilitate a direct comparison of the size of two partial correlations, they were tested using a parametric bootstrapping approach. The estimates and their respective standard errors were computed in Mplus, while the bootstrapping procedure was implemented in R. This approach ensured that neither the deviation from normality nor the lack of independence between participants biased the results and revealed that the partial correlations of the same facets of normative beliefs and aggression were significantly higher than the correlations between different facets of two constructs at all four data waves.

**Table 2:** Standardized partial correlations between physical and relational aggression with corresponding and non-corresponding norm facets.

	T1	T2	T3	T4
Physical aggression – physical norms	0.47***	0.51***	0.46***	0.45***
Physical aggression – relational norms	0.06*	0.08*	0.05	0.11**
Relational aggression – relational norms	0.36***	0.42***	0.43***	0.47***
Relational aggression – physical norms	0.09**	0.15***	0.06	0.10*

*p < 0.05, **p < 0.01, ***p < 0.001.

**FIGURE 3:**
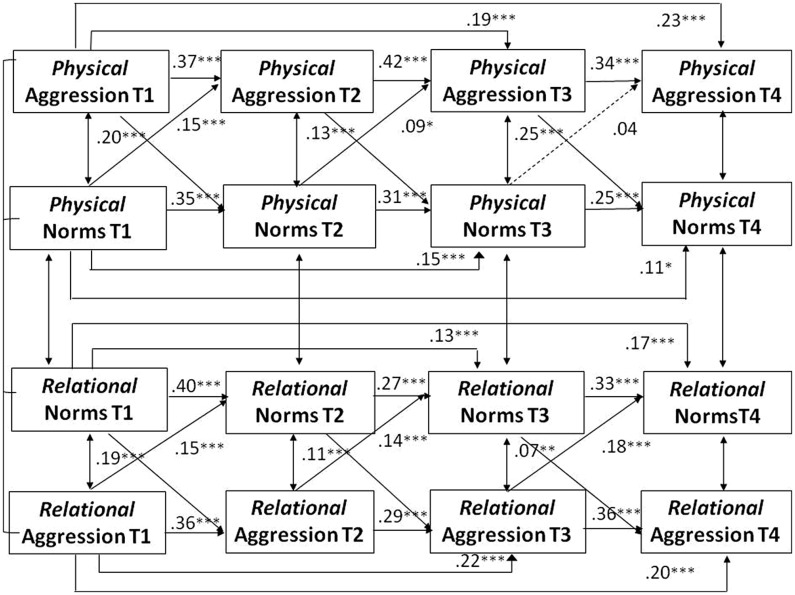
**Boys’ paths of physical and relational aggression and normative approval over four data waves.** *N* = 892. Standardized coefficients are shown. All model variables controlled for year cohort, ethnic background, and school type. Coefficients for the cross-sectional associations included in the model are shown in **Table 3**. Broken lines indicate non-significant paths.

**FIGURE 4:**
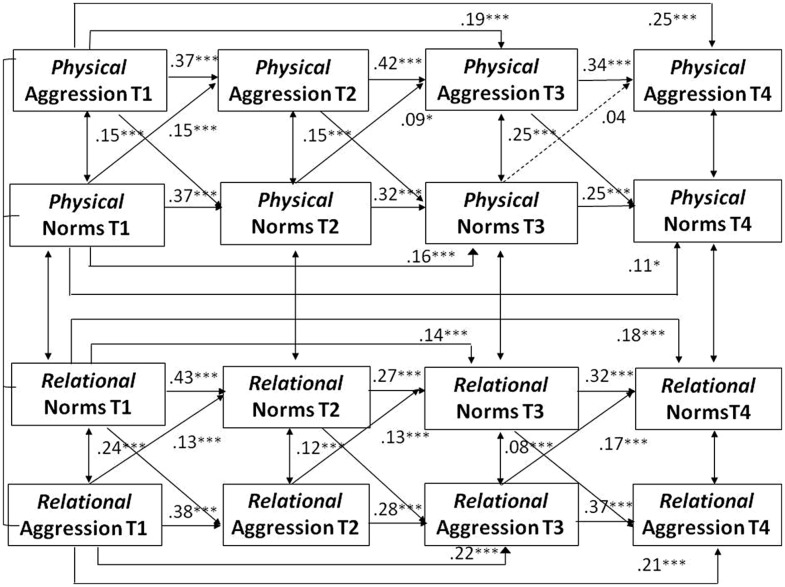
**Girls’ paths of physical and relational aggression and normative approval over four data waves.** *N* = 962. Standardized coefficients are shown. All model variables controlled for year cohort, ethnic background, and school type. Coefficients for the cross-sectional associations included in the model are shown in **Table 3**. Broken lines indicate non-significant paths.

Hypothesis 5 proposed a reciprocal influence between norms and behavior over time. This prediction was examined through cross-lagged path analyses in which physical and relational aggression were related to the corresponding normative beliefs over the four data waves. We assumed that despite gender differences in the mean levels of aggressive behavior and its normative approval, the longitudinal links between aggressive norms and the two forms of aggressive behavior would be the same for both gender groups. Therefore, we began by testing a multi-group model in which the stability paths as well as the cross-lagged paths were constrained to be equal for boys and girls. Year cohort, school type, and ethnic background were again included as covariates. This constrained model fitted the data well, χ^2^ (df = 172) = 412.59, *p* = 0.001, CFI = 0.96; TLI = 0.93, SRMR = 0.06, RMSEA = 0.04, 95% CI 0.03–0.04). In a second step, the constrained model was compared with an unconstrained model in which all paths were allowed to vary between the two gender groups. The χ^2^ difference test indicated that the unconstrained model did not fit the data better than the constrained model, χ^2^ difference (df = 28) = 1.66, *p* = 0.76. Therefore, the more parsimonious constrained model was adopted as the final model for evaluating Hypothesis 5. In the constrained model, the unstandardized path coefficients for the cross-lagged and stability paths are identical for boys and girls, whereas the standardized coefficients may vary because they take differences in the error variance in each gender group into account. To facilitate comparability of our findings with previous studies, we present the standardized paths for boys and girls in **Figures 3** and **4**. For clarity of presentation, the two figures only present the longitudinal paths. The cross-sectional associations at each of the four data waves, controlling for the covariates, are presented in **Table 3**.

**Table 3:** Standardized cross–sectional associations in the path models presented in **Figures 3** and **4**.

	T1		T2		T3		T3	
	Boys	Girls	Boys	Girls	Boys	Girls	Boys	Girls
Physical aggression – relational aggression	0.58	0.50	0.41	0.32	0.35	0.23	0.42	0.37
Physical aggression – physical norms	0.52	0.44	0.33	0.25	0.32	0.11^a^	0.15^a^	0.28
Physical norms – relational norms	0.48	0.55	0.21	0.33	0.28	0.37	0.28	0.28
Relational aggression – relational norms	0.42	0.47	0.26	0.30	0.28	0.32	0.31	0.23

^a^*p* < 0.05, all other correlations significant at *p* < 0.001. Controlled for year cohort, ethnic background, and school type.

Both measures of aggressive behavior and both normative approval measures showed significant, yet moderate stabilities across the four data waves, with stability coefficients between adjacent data waves ranging from 0.27 to 0.43. Significant cross-lagged paths were found for both gender groups between the normative acceptance of relational aggression and relationally aggressive behavior from T1 to T4. The more participants considered relational aggression as acceptable, the more relationally aggressive behavior they showed at the subsequent data wave. The indirect effects, shown in **Table 4**, revealed that normative beliefs at T1 predicted aggressive behavior at T4 through both norms and behavior at T2 and T3.

**Table 4:** Significant indirect effects from the path models in **Figures 3** and **4**.

	Boys	Girls
		
**Norms to behavior**		
Phy Norms T1 → Phy Agg T2 → Phy Agg T3 → Phy Agg T4	0.019	0.021
Phy Norms T1 → Phy Norms T2 → Phy Agg T3 → Phy Agg T4	0.010	0.011
Rel Norms T1 → Rel Agg T2 → Rel Agg T3 → Rel Agg T4	0.020	0.024
Rel Norms T1 → Rel Norms T2 → Rel Agg T3 → Rel Agg T4	0.002	0.002
Rel Norms T1 → Rel Norms T2 → Rel Norms T3 → Rel Agg T4	0.008	0.009
**Rel Norms T1**→** Rel Agg T2**→** Rel Norms T3**→** Rel Agg T4**	**0.002**	**0.002**
**Behavior to norms**		
Phy Agg T1 –> Phy Agg T2 –> Phy Agg T3 → Phy Norms T4	0.034	0.035
Phy Agg T1 → Phy Agg T2 → Phy Norms T3 → Phy Norms T4	0.013	0.014
Phy Agg T1 → Phy Norms T2 → Phy Norms T3 → Phy Norms T4	0.015	0.015
**Phy Agg T1**→** Phy Norms T2**→** Phy Agg T3**→** Phy Norms T4**	**0.004**	**0.004**
Rel Agg T1 → Rel Norms T2 → Rel Norms T3 → Rel Norms T4	0.013	0.012
Rel Agg T1 → Rel Agg T2 → Rel Norms T3 → Rel Norms T4	0.017	0.015
Rel Agg T1 → Rel Agg T2 → Rel AggT3 → Rel Norms T4	0.019	0.018
**Rel Agg T1**→** Rel Norms T2**→** Rel Agg T3**→** Rel Norms T4**	**0.003**	**0.003**

*Phy Agg, Physically aggressive behavior; Rel Agg, relationally aggressive behavior; Phy Norms, normative approval of physical aggression; Rel Norms, normative approval of relational aggression. None of the confidence intervals of the displayed coefficients included zero. Effects highlighted in bold indicate the reciprocal pathways.*

A similar pattern emerged for physical aggression. The prospective paths from the normative approval of physical aggression to aggressive behavior were significant from T1 to T2 and from T2 to T3, but not for the final period from T3 to T4. Two significant indirect effects emerged for physical aggression: the normative approval of aggression at T1 had an indirect effect on T4 levels of aggression through its impact on aggressive behavior at T2 and T3 and through the impact on T2 normative approval and T3 aggression (see **Table 4**). A full reciprocal pathway from norms to behavior, as indicated by indirect effects from T1 normative beliefs to T4 behavior through T2 aggressive behavior and T3 normative beliefs, was found for relational aggression, but not for physical aggression.

In addition to the paths from norms to behavior, significant pathways were also found from aggressive behavior to normative beliefs from T1 to T4 for both gender groups. The two forms of aggression predicted T4 normative beliefs indirectly from T1 via both normative beliefs and behavior at T2 and T3. Normative approval of aggression not only predicted later aggressive behavior, but engaging in more aggression also predicted greater normative approval of aggression over time. There were significant reciprocal pathways from T1 aggression to T2 normative beliefs to T3 aggressive behavior to T4 normative beliefs for both physical and relational aggression, as shown in **Table 4**. The more aggressive participants were initially, the more they endorsed aggression as normative at the second data wave. This greater normative approval predicted higher aggressive behavior at the third data wave which, in turn, was predictive of greater normative approval at the fourth and final data wave. These findings confirm the mutually reinforcing influence of norms and behavior that was predicted in Hypothesis 5. Although some of the cross-lagged associations between norms and behavior differed in magnitude for physical and relational aggression, none of the differences reached statistical significance.

## DISCUSSION

The present study was designed to examine patterns of change in physical and relational aggression over three years in the course of adolescence, focusing on the moderating role of gender and the mediating role of normative beliefs. A large sample of adolescents representing the full range of the secondary school system provided measures of physical and relational aggression and of the normative approval of both forms of aggression at four data waves separated by 12-month intervals.

Across the four data waves, physical and relational aggression were significantly, but moderately correlated. This finding, which is consistent with evidence by Lansford et al. (2012) that found correlations of similar magnitude in nine countries, suggests two conclusions. The first is that the two forms of aggression tend to co-occur in an individual’s behavioral repertoire, supporting the view that they share the underlying motivation to harm. The second is that they capture distinct forms of behavior, in line with the conceptualization of physical and relational aggression as representing different “vehicles of harm” that carry distinct normative evaluations (Crick et al., 2007). This latter conclusion is corroborated by the finding that the two forms of aggression showed different patterns of change during adolescence. Whereas physical aggression decreased, relational aggression increased over the 3-year period.

Consistent gender differences were found for physical aggression, with boys scoring higher than girls. Boys also had higher means than did girls on relational aggression, but the significant gender by aggression form interaction showed that the gender difference was smaller than for physical aggression. This finding for adolescents in Germany is in line with previous research from other countries (e.g., Lansford et al., 2012; Tseng et al., 2013) as well as meta-analytic reviews (Card et al., 2008; Scheithauer et al., 2008). Few studies have examined gender differences in normative beliefs about physical and relational aggression. Werner and Hill (2010) found that boys were more approving of both forms of aggression than were girls, but their sample was younger than the participants in our study. In the present study, the comparison of the simple means suggested that boys were more approving of both physical and relational aggression across all four data waves, but the intercept-slope models including both facets of aggression as well as the covariates showed that the gender difference held only for the approval of physical aggression. This pattern matches the interaction of gender and aggression form that was found for aggressive behavior and further supports the conceptual association between normative beliefs and behavior. The significant interaction of gender and time indicated that across both types of normative beliefs, boys showed a larger decrease in the course of adolescence than did girls. One possible explanation might be that boys are later to embark on the downward trend for the approval of aggression than are girls. Gender differences in maturation that have been found consistently during adolescence and linked to differences in brain development may also account for boys’ delayed rejection of aggression as a normatively accepted pattern of behavior (Silberman and Snarey, 1993). For example, a longitudinal study on empathy that followed adolescents from the age of 13 to the age of 18 found that although cognitive perspective taking increased in both gender groups, the increase started later for boys than for girls (Van der Graaff et al., 2014). Another possibility might be that through the increase in cross-gender interactions in adolescence, including romantic relationships, girls may socialize boys away from the approval of aggressive behavior. According to the “two cultures” perspective, boys and girls grow up in largely gender-segregated peer groups (Maccoby, 1998), which start coming together in adolescence so that boys would have greater exposure to female norms regarding aggressive behavior. However, whether changes in boys’ or girls’ normative beliefs and aggressive behavior may be linked to their experiences with the opposite sex in friendships and romantic relationships is a question for future research (Underwood and Rosen, 2009).

Despite the gender differences in the levels of both forms of aggression, the cross-lagged paths between aggressive behavior and normative beliefs did not vary by gender. This finding is in line with previous research with adolescents in the United States based on both self-reports (Werner and Nixon, 2005) and peer nominations (e.g., Kawabata et al., 2014) of aggression, which also found no evidence of gender differences in the *relationship* between normative beliefs and aggressive behavior. It supports the generality of social information processing models of aggressive behavior that assign a central role to the interplay between normative beliefs and aggressive behavior (Huesmann and Kirwil, 2007). In their critical examination of research on direct and indirect aggression in childhood, Underwood et al. (2001, p. 260) reasoned that “the best understanding of aggression among girls might require different conceptual frameworks […] than those that have been used with boys.” However, the present findings suggest that the psychological processes assumed to connect normative beliefs and aggressive behavior may operate in a similar way in boys and girls.

Regarding the directional pathways of norms and behavior, we predicted a mutually reinforcing cycle of both constructs over time. This pattern was confirmed with regard to relational aggression. The higher the approval of relational aggression, the higher the scores on the subsequent measure of relational aggression, and the higher the aggressive behavior, the greater the normative approval of this form of aggression at the next data wave, controlling for the stability of normative beliefs and behavior. These findings are consistent with the “individual systems” model by Fontaine and Dodge (2009), which proposes that individuals’ aggressive behavior influences their subsequent evaluation of behavioral options. They are also compatible with classic social psychological theories that highlight the impact of behavior on beliefs and attitudes. These models, such as Festinger’s (1957) theory of cognitive dissonance and Bem’s (1972) self-perception theory, assume that individuals use their behavior as a source of information from which to draw inferences about their own attitudes and beliefs. In this vein, the more aggressive behavior individuals show, the more they come to think that they must approve of this form of behavior. For physical aggression, the pattern was somewhat less consistent, as no path from normative beliefs at T3 to physical aggression at T4 was found. The latter finding was similar to evidence by Fontaine et al. (2009) for a measure of antisocial behavior that included acts of physical aggression. They found that aggressive social cognitions in grade 8 were unrelated to antisocial behavior in grade 11, whereas antisocial behavior in grade 8 significantly predicted aggressive social cognitions in grade 11. One possible explanation for the lack of a significant path from normative beliefs to physical aggression at the final data wave of our study could be that the normative acceptance of physical aggression was not only substantially lower than the acceptance of relational aggression at each point in time but also showed a significantly greater decrease over the four data waves, which may have reduced its impact on behavior. Further research is needed to explore this possibility.

### STRENGTHS AND LIMITATIONS

The present study has several strengths. It followed a large sample of adolescents from schools varying in academic orientation at four data waves over three years. It collected information not only about two forms of aggression, physical and relational, that have been distinguished in the debate about gender differences in aggression, but also broke down the measurement of normative beliefs into the approval of physical and relational aggression. This made it possible to examine the specificity of the link between norms and behavior within each form of aggression, contributing to the conceptual development of the distinction between physical and relational aggression and their normative foundations. In addition, the four-wave design of our study facilitated the analysis of the mutual reinforcement of normative beliefs and behavior, providing evidence that aggressive behavior is not only influenced by the normative approval of this form of antisocial behavior but also influences subsequent normative beliefs that, in turn, promote further aggression.

The main limitation of the present study is the reliance on self-reports to measure both physical and relational aggression. Social desirability concerns may have affected reports of both aggressive behavior and normative approval of aggression, which could have inflated the correspondence between the two constructs. This possibility, which is shared by other studies linking normative beliefs to self-reported aggression (e.g., Werner and Nixon, 2005; Burton et al., 2013), calls for future research using peer nominations or teacher reports to assess physical and relational aggression in adolescence. A second limitation is the age range from early to middle adolescence. Although the 3-year design of our study covered an extensive and critical time window in adolescent development, using a wider time span from childhood onward could further clarify the trajectories of normative beliefs and behavior for both physical and relational aggression, including the possibility of detecting nonlinear patterns of change.

## CONCLUSION

The present longitudinal study following adolescents from age 13 to age 16 has provided further support for the conceptual distinction between physical and relational aggression. The two forms of aggression were found to be differentially related to gender, to be linked to specific normative beliefs, and to show different patterns of change in the course of adolescence. Our findings suggest that the age-normative decline of aggression from childhood to adolescence may be true primarily for physical aggression and may be accompanied by an increase in relational aggression. They also suggest that the patterns of change as well as the psychological pathways linking norms and behaviors may be similar for adolescent boys and girls despite gender differences in the levels of physical and relational aggression.

## Conflict of Interest Statement

The authors declare that the research was conducted in the absence of any commercial or financial relationships that could be construed as a potential conflict of interest.
